# Impostor phenomenon and ambiguity tolerance in practicing physical therapists: an exploratory correlational study

**DOI:** 10.5116/ijme.6532.4c20

**Published:** 2023-11-10

**Authors:** Melissa Carroll, Sean Griech

**Affiliations:** 1 Department of Anatomy and Cell Biology, School of Medicine and Health Sciences, George Washington University, USA; 2 Division of Healthcare Professions, Doctor of Physical Therapy Program, DeSales University, USA

**Keywords:** Impostor syndrome, impostorism, tolerance to ambiguity, professional burnout

## Abstract

**Objectives:**

Investigate the prevalence and contextualize the relationship of impostor phenomenon (IP) and ambiguity tolerance (AT) in practicing physical therapists (PTs).

**Methods:**

Online survey including demographic questions, Clance Impostor Phenomenon Scale (CIPS), and Tolerance of Ambiguity Scale (TAS). Descriptive analyses assessed (N = 422) demographic data, CIPS, and TAS scores. Chi-square tests determined distribution across demographic variables.  Kruskal-Wallis tests assessed differences between CIPS and TAS.  Age was a proxy for career stage in Pearson product-moment correlations to assess relationships between CIPS and TAS.

**Results:**

In practicing PTs (M age = 42.12, SD = 12.34), moderate (48.6%; n = 205) to frequent (26.8%; n = 113) IP feelings were prevalent, but only 31.5% (n = 133) were true impostors.  Significant differences exist in clinical experience for CIPS, H(7, n = 422) = 67.82, p <.001 and TAS, H(7, n = 422) = 21.79, p= .003. Most PTs tolerate ambiguity (M = 54.93, SD = 8.19).  A moderate negative correlation between age and CIPS, r = -.36, p <.001 and a small negative correlation between age and TAS, r=-.19, p <.001 exists. Age accounts for 13% of the variance per IP and 3.6% variance per TAS score.  A small positive relationship exists between CIPS and TAS, r = .10, p <.05.

**Conclusions:**

Practicing PTs experience moderate to frequent IP and are ambiguity tolerant.  Clinical experience is inversely related to IP and AT.  Almost half of early-career PTs feel like impostors, which can lead to decreased job satisfaction, burnout, psychological distress, feelings of self-doubt, and depression.

## Introduction

The term impostor phenomenon (IP) describes an experience of intellectual phoniness.^1^^-^^3^ Research uses IP, “impostorism,” and “impostor syndrome” synonymously; however, it is critical to recognize that IP is a psychological construct rather than a diagnostic criterion. People experiencing IP will attribute achievements to chance and often feel distress over being evaluated for fear of being exposed as a fraud.^2^^-^^5^ Within healthcare research, IP has been of particular interest partly due to the daily complexity and ambiguity healthcare workers face.^5^^-^^9^ Physical therapists (PTs) are no exception, especially considering the lack of standardization and wide range of clinical settings or practice environments. As a result, fraudulent feelings may emerge concerning clinical skills and abilities, or as self-doubt and mistrust of professional expertise.^1^^,^^4^^,^^5^   These IP feelings may persist despite experience, education, or achievements.

Data are conflicting regarding feelings of IP in dichotomized gender (men and women),^4^^,^^10^^,^^11^ but explicit research across the gender spectrum (to include transgender, non-binary, or other self-identified categories) is lacking.  Clance and Imes initially focused on the belief that women were more affected by IP characteristics.^2^ More recently, this long-standing belief has been clarified, stating that IP in women may stem from society’s gender stereotypes and bias towards male accomplishments.^10^^-^^12^ For some healthcare students, residents, and clinicians, women scored significantly higher on the Clance Impostor Phenomenon Scale (CIPS), however there were no statistical differences within leadership or managerial positions.^5^^,^^9^^,^^11^^,^^13^^,^^14^

There is also conflicting evidence regarding the stability or development of IP feelings as medical students and residents progress in their professional or specialty training.^9^^, ^^14^^-^^16^ These results are supported by the idea that most people will have one IP episode at some point in their professional careers.^2^^,^^17^  A recent scoping review identified IP rates between 22-60% among medical students and 33-44% among North American, South Asian, Southeast Asian, Middle Eastern, and West African medical residents.^6^ However, evidence supports that 30.2% of healthcare (medical, dental, nursing, and pharmacy)^7^ and 39% of physician assistant students^18^ exhibit frequent to intense IP characteristics.  Only moderate IP feelings have been reported for Doctor of Physical Therapy (DPT) students, but the scores were from a small sample (n = 54) and ranged from a few to intense IP characteristics.^19^ In a Doctor of Chiropractic program, rates of IP experiences did not correlate with the academic year.^13^ Therefore, a question remains regarding the association between academic or postgraduate years and IP experiences in healthcare.

Ethnicity has also been a factor in IP research, with increased discriminatory experiences predicting higher IP levels and feelings of incompetence.^20^ In 2020, a systematic review demonstrated that IP experiences were higher among ethnic minority groups. Feelings of IP were inversely correlated with psychological well-being and were predictive of anxiety in some groups.^1^^,^^4^ One study reported that White and Asian medical students exhibit less IP than other ethnicities, but this study did not use the CIPS to determine the IP rates.^17^

The lack of evidence surrounding the prevalence of IP in practicing PTs highlights a knowledge gap.  PTs practice autonomously; the plan of care may not have specific steps or protocols to follow, leading to significant variability while adapting interventions to the individuals they treat.  Furthermore, PTs often encounter scenarios in which they must adapt to unforeseen circumstances involving a lack of time or resources, decreased patient tolerance or function, and increased productivity standards.  These complex, novel, and unsolvable clinical scenarios require different levels of ambiguity tolerance (AT) and may be daunting or threatening to some PTs – especially those who already consider themselves impostors.

An individual’s perception of and response to unfamiliar situations, concepts, or environments determines their AT.^21^^-^^25^ Someone with AT will perceive new or unknown scenarios openly rather than as a threat.  This can be a valuable characteristic for clinical practice, especially when faced with increased unpredictability in more complex settings.^21^^,^^22^^,^^25^^,^^26^ Although AT is widely believed to be a personality trait, it has been identified and quantified for research.^21^   Similar to IP, dichotomous gender does seem to be a controversial factor of AT, but differences seem dependent on the situational context.^27^ In medical students, men were more tolerant regarding openness to new experiences, while women demonstrated better AT with social conflicts and insoluble problems; however, an uncommon AT outcome measure was used.^22^^,^^27^  Reports also indicate no AT gender differences in practicing physicians.^23^ Yet, ethnicity appears to influence the degree of AT within medicine.  Asian individuals reported the lowest AT based on the tolerance of ambiguity scale (TAS), while there was no difference among White, Hispanic, or Black individuals.^23^ Recent evidence suggests that DPT students are comparably as tolerant as medical students when managing ambiguous situations.^28^

It seems plausible that being classified as an impostor or having frequent to intense IP characteristics may increase in more ambiguous clinical situations.  While similarities exist between IP and AT, a relationship between the two has not been explored in physical therapy.  The practice environment can often be very stressful and unpredictable due to the nature of the patient population and working within a multidisciplinary healthcare team. Little is known about factors that influence or lead to feelings of IP in PTs; however, a therapist’s response to uncertain or ambiguous situations, concepts, or workplaces may contribute.  Therefore, an ability to deal with ambiguous situations may help PTs decrease IP feelings.

The purpose of this study was to determine the prevalence of IP and AT among practicing PTs and explore whether factors of self-identified gender, age, ethnicity, highest degree earned, geographical location, clinical experience, or clinical setting influence the expression of IP or AT characteristics.  Additionally, this study aimed to explore if there were predictive or explanatory values between IP and AT.

It was expected that most practicing PTs would experience moderate feelings of IP and demonstrate a tolerance to ambiguity.  It was also hypothesized that there would be no significant differences regarding IP or AT due to geographical locations, however, there would be significant differences due to gender identification, ethnicity, degrees earned, and clinical experience, similar to findings in other healthcare professions.  It was also assumed that PTs dealing with a higher percentage of medically complex patients (i.e., acute care, skilled nursing facilities, and inpatient rehabilitation settings) would have more AT.

Identifying IP and AT characteristics in practicing PTs allows future research to investigate risks for burnout, anxiety, and depression which could lead to reduced treatment quality for patients.  Our research is essential for a foundational interpretation of IP and AT characteristics in practicing PTs in the United States due to the paucity of evidence regarding physical therapists.

## Methods

### Research Design and Participants 

This was an exploratory correlational study to describe the levels of IP and AT in practicing PTs, and to contextualize the relationship between each psychological construct. An online survey was deployed and open for three months (March - May 2021).  Participation was voluntary and informed consent was received through clicking “I agree” to a consent statement before accessing the survey.  Participants were only included in the study if they were currently practicing PTs – this was the sole inclusion criteria.  Skip logic was added to the survey to remove participants that did not meet the inclusion criteria. The university institutional review board approved this study.

### Sampling Method

Participants were identified and recruited through several American Physical Therapy Association (APTA) state chapters (Pennsylvania, New York, Maryland, and the District of Columbia).  Additionally, APTA Academy of Orthopaedic Physical Therapy (AOPT) members who opted to receive surveys were also recruited through an electronic invitation.  Lastly, social media platforms were used to recruit participants through an open link to the survey.  Participants were also encouraged to forward the survey link to practicing PTs within their networks to improve the survey’s reach.

#### Instrument

The online survey consisted of 36 questions.  Demographic information was collected to provide context regarding gender identification, ethnicity, degrees earned, current employment status, clinical setting, geographical location, and length in clinical practice (an indication of clinical experience regardless of different clinical settings across an entire career).

#### Impostor Phenomenon

Use of the Clance Impostor Phenomenon Scale (CIPS) was granted by the developer Dr. Pauline Rose Clance (personal communication).  The CIPS is a 20-item Likert scale that asks respondents to self-identify with statements on a range from “Not at all” (1 point) to “Very true” (5 points).^29^ The maximum score is 100, and the minimum score is 20.  Respondents are classified as having few impostor characteristics if the total score is 40 or less, moderate if the score is between 41 and 60, frequent if the score is between 61 and 80, and intense impostor characteristics if the score is higher than 80.  To distinguish impostors from non-impostors, a cutoff score of 62 has been recommended.^30^ Three subscales have been indicated within the IP construct: fake (Q#6,13), luck (Q#5,11), and discount (Q#15,19),^30^^,^^31^ however, most evidence concludes that the one-factor model is the best fit.^32^

#### Ambiguity Tolerance 

The Tolerance for Ambiguity Scale (TAS) was used to measure AT with a sliding scale ranging from 16 to 112, with lower scores indicating ambiguity tolerance.^21^ The TAS is a 16-item scale with seven Likert-type responses to each statement ranging from “Strongly Disagree” to “Strongly Agree.” According to the original TAS, ambiguity exists within three main situations: novelty (Q#2, 9, 11, 13), complexity (Q#4, 5, 6, 7, 8, 10, 14, 15, 16), and insolubility (Q#1, 3, 12).  Reports on the internal consistency indicate flaws within Budner’s original design (⍺ = 0.49); however, it is still the most widely used scale to measure AT.^22^^, ^^33^  There is no established cutoff score or strict interpretation of the raw TAS scores; however, prior studies indicate healthcare providers that fall between 44 - 52 are tolerant of ambiguity.^25^^,^^34^  Budner also used the median score to divide respondents into tolerant (below the median) or intolerant (above the median).^21^

### Data Collection 

The survey was piloted to ensure appropriate data collection, assess the clarity of survey questions (options provided, grammar, and vocabulary), and analyze the internal consistency of the CIPS and TAS within the target population.  Recent DPT graduates received an invitation to participate two months before distributing the final survey.  The pilot survey was open for approximately one week and was completed by 21 participants.  Based on the pilot data and feedback, a gender option was added to include “non-binary”.  Furthermore, an additional yes or no question (“Are you currently practicing as a physical therapist?”) was added as a second verification that participants met the inclusion criteria.  Lastly, a decision was made to exclude incomplete survey responses. The pilot data were assessed for Cronbach’s alpha coefficients for the CIPS, ⍺ = .91, 95% CI [.84, .96] and TAS, ⍺ = .68, 95% CI [.43, .85].   The CIPS mean score was 57.33 (SD =13.55) and the TAS mean score was 55.52 (SD=10.19).  The outcome measures were randomized during administration to reduce survey fatigue, with approximately half of the participants receiving the CIPS first, followed by the TAS, and vice versa.

### Data Analysis

Pilot data was not included in the final analysis.  Nominal and ordinal data were transformed and coded for analysis by IBM® SPSS® Statistics software version 27.  When appropriate, data were tested for normality using the Kolmogorov–Smirnov (K-S) tests.  Descriptive analyses were conducted for the demographic data, the raw CIPS, and TAS scores.  Chi-square tests were used to assess the distribution of respondents across the demographic variables and the difference between group CIPS and TAS scores were assessed with Kruskal-Wallis tests.   Length in clinical practice was used to classify early (0-10 years), middle (11-20 years), and late-career stage (more than 21 years).^35^ Kruskal-Wallis tests were conducted to determine if there were significant differences between career stages and CIPS and TAS scores.  Age was used as a quantitative proxy variable for respondents’ career stage; Pearson product-moment correlations were used to determine any relationship between age and the CIPS or TAS Scores.  Finally, a bivariate regression analysis was performed to assess the influence of CIPS on TAS Scores.

## Results

A total of 497 individuals responded to the survey.  Of the respondents who completed the entire survey, 84.9% indicated that they were practicing PTs, resulting in 422 respondents included in the final analysis.  The respondents’ ages ranged from 23 - 74, with a mean of 42.12 (SD = 12.34) years.  Most respondents identified as women (61.8%; n = 261), White (86%; n = 363), employed full-time (74.9%; n = 316), earned a DPT degree (56.6%; n = 239) and had greater than ten years of clinical practice experience (59%; n = 249).  Although respondents represented each geographical location and each clinical setting,^36^ there was a statistically significant difference in representation from each location. χ^2^(9, 422) = 196.8, p<.001 and setting, χ^2^(10, 422) = 1068.5, p<.001(Table 1).

### Impostor Phenomenon

The distribution of respondents’ scores for the CIPS was approximately normal.  The CIPS scores ranged from 22 - 97, with a mean of 55.52 (SD = 15.18).  The most prevalent IP characteristics were moderate (48.6%; n = 205) to frequent (26.8%; n = 113) experiences.  However, based on the previously established cutoff score (>62), only 31.5% (n = 133) of the sample population were classified as impostors^30^ (Table 1).

To compare CIPS scores between the demographic variables, the data were first tested for normality and equal variances using K-S tests.  Assumptions were not met, so Kruskal-Wallis tests were used.  There were no significant differences in CIPS scores between geographical location, clinical setting, ethnicity, and gender.  However, there was a significant difference in IP experiences between degrees earned, H(5, n = 422) = 34.19, p<.001, employment status, H(2, n=422) = 6.28, p=.04, and clinical experience, H(7, n=422)=67.82, p<.001. (Table 1) Post hoc analyses were conducted using a Dunn-Bonferroni approach for each significant Kruskal-Wallis test.  The CIPS score was significantly higher for PTs who earned a DPT alone (M=59.05) than a Bachelor of Science in Physical Therapy (BSPT), M = 48.00, p<.01, Master of Physical Therapy (MPT), M = 50.53, p<.01, and multiple degrees, M = 50.58, p< .001.  CIPS score for full-time employment was also significantly higher (M = 56.18) than part-time, M= 51.92, p<.05.  The clinical experience post hoc analyses, adjusted by the Bonferroni correction, revealed several significant differences, with the CIPS score significantly higher in PTs with less than one year experience (M = 71.04) than 6-10 years, M = 58.50, p = .011, 11-15 years, M = 56.31, p < .01, 16-20 years, M = 55.70, p < .01, 21-30 years, M = 50.86, p< .001, and over 31 years, M = 47.10, p < .001.  Furthermore, CIPS scores were significantly lower for PTs with 21-30 years (M = 50.86) than for 1-3 years, M = 59.64, p = .013 and 4-5 years, M = 59.19, p = .050.  Lastly, CIPS scores were significantly lower for PTs with over 31 years of experience (M = 47.10) than 1-3 years, M = 59.64, p < .001, 4-5 years, M = 59.19, p = .001, 6-10 years, M = 58.50, p<.01 and 11-15 years, M = 56.31, p = .02.

### Ambiguity Tolerance

The distribution of respondents’ scores for the TAS was approximately normal.  The TAS scores ranged from 28 - 81, with a mean of 54.91 (SD=8.21).  To compare TAS scores between the demographic variables, the data were first tested for normality and equal variances using K-S tests.  Assumptions were not met so Kruskal-Wallis tests were used.  There were no significant differences between geographical location, clinical setting, ethnicity, and gender in TAS scores.  However, there was a significant difference between clinical experience and TAS, H(7, n = 422) = 21.79, p = .003, with a mean rank TAS score of 256.26 for 6-10 years, 238.63 for 1-3 years, 231.65 for less than one year, 219.44 for 21-30 years, 203.56 for 16-20 years, 198.89 for 4-5 years, 178.62 for over 31 years, and 176.09 for 11-15 years.  The TAS scores were significantly higher in PTs with 6-10 years of experience (M = 57.91) than for 11-16 years, M = 52.76, p = .015 and over 31 years, M = 52.12, p = .009.

To assess ambiguity tolerance in clinical settings, respondent data were compressed into three groups: Outpatient (hospital and private), Inpatient (acute care, skilled nursing facility, and inpatient rehabilitation), and Other.  Following Budner’s original analysis, each group was assessed against the median to determine ambiguity tolerance (below) versus intolerance (above).^21^ The median TAS score was 55 (28 – 81) across all respondents, 44% (n = 146) of the outpatient group were below the median compared to 46% (n = 18) of the inpatient and 54% (n = 28) of the other group.

### Career Stage, IP, and AT

To explore the relationships between career stage and the psychological constructs it was decided to categorize respondents as early, middle, or late-career PTs using length in clinical practice.  Consequently, there were statistically significant age differences between the career groups, H(2, n = 422) = 337.75, p<.001(Table 1).  There was also a significant association between the degrees earned and the career stage, χ^2^(10, 422) = 301.84, p<.001. As expected, late-career PTs were statistically more likely to have earned a BSPT, MPT, Transitional Doctor of Physical Therapy (tDPT), or multiple degrees than early-career PTs who were more likely to have earned a DPT degree. (Table 1) Early-career PTs were also more likely to be employed full-time when compared to middle or late-career PTs, χ^2^(4, 422) = 20.78, p<.001 (Table 1).

**Table 1:** Demographics (N=422)

**Characteristics**	All Respondents	CIPS score	p-value^a^	TAS Score	p-value^a^	Career Stages		
						Early	Middle	Late
	N = 422	M (SD)		M (SD)		n = 173	n = 95	n = 154
**Age,** mean (SD)	42.12 (12.3)	-		-		30.99 (4.8)	40.64 (4.2)	55.55 (7.7)
**Gender,** no. (%)^								
Male	159 (37.7)	54.01 (14.1)	.26	54.69 (8.7)	.82	60 (34.7)	42 (44.2)	57 (37.0)
Female	261 (61.8)	56.54 (15.8)		55.07 (7.9)		113 (65.3)	53 (55.8)	95 (61.7)
Non-binary	1 (.2)					-	-	1 (.6)
Prefer not to answer	1 (.2)					-	-	1 (.6)
**Ethnicity**, no. (%)								
White	363 (86.0)	55.58 (15.2)	.85	55.07 (8.3)	.11	145 (83.8)	79 (83.2)	139 (90.3)
Hispanic, Latino, or Spanish origin	8 (1.9)	49.25 (12.4)		56.63 (5.8)		2 (1.2)	3 (3.2)	3 (1.9)
Black or African American	7 (1.7)	54.00 (17.7)		52.71 (7.7)		5 (2.9)	1 (1.1)	1 (.6)
Asian	17 (4.0)	54.76 (14.8)		57.53 (8.3)		8 (4.6)	7 (7.4)	2 (1.3)
American Indian or Alaska Native	-	-		-		-	-	-
Middle Eastern or North African	-	-		-		-	-	-
Native Hawaiian or Other Pacific Islander	-	-		-		-	-	-
Mixed/More than one	14 (3.3)	56.79 (15.5)		49.71 (6.7)		9 (5.2)	3 (3.2)	2 (1.3)
Other/Unknown	2 (.5)	61.50 (7.8)		48.00 (4.2)		1 (.6)	1 (1.1)	-
Prefer not to answer	11 (2.6)	57.27 (17.2)		53.91 (7.8)		3 (1.7)	1 (1.1)	7 (4.5)
**Degrees Earned**, no. (%)^#^								
Physical Therapy Assistant	4 (.9)					-	2 (2.1)	2 (1.3)
Bachelor of Science in Physical Therapy	78 (18.5)	48.00 (14.8)	<.001	54.08 (9.0)	.21	1 (.6)	5 (5.3)	72 (46.8)
Master of Physical Therapy	85 (20.1)	50.53 (15.1)		54.08 (7.3)		-	23 (24.2)	62 (40.3)
Transitional Doctor of Physical Therapy	86 (20.4)	55.21 (14.0)		55.54 (6.9)		1 (.6)	23 (24.2)	62 (40.3)
Doctor of Physical Therapy	239 (56.6)	59.05 (14.7)		56.00 (8.1)		169 (97.7)	56 (58.9)	14 (9.1)
DPT with PhD and/or Equivalent	24 (5.7)	58.60 (18.6)		51.10 (9.1)		3 (1.7)	6 (6.3)	15 (9.7)
Other	25 (5.9)					2 (1.2)	3 (3.2)	20 (13.0)
**Employment Status**, no. (%)								
Full-Time	316 (74.9)	56.18 (14.9)	.04	55.32 (8.0)	.47	148 (85.5)	69 (72.6)	99 (64.3)
Part-Time	76 (18.0)	51.92 (15.6)		53.47 (9.2)		18 (10.4)	17 (17.9)	41 (26.6)
Per-diem	30 (7.1)	57.67 (15.9)		54.33 (7.3)		7 (4.0)	9 (9.5)	14 (9.1)
**Years in Clinical Practice**, no. (%)								
<1	27 (6.4)	71.04 (11.3)	<.001	56.07 (7.9)	.003	27 (15.6)	-	-
1-3	53 (12.6)	59.64 (13.6)		57.09 (8.4)		53 (30.6)	-	-
4-5	37 (8.8)	59.19 (11.6)		54.68 (7.9)		37 (21.4)	-	-
6-10	56 (13.3)	58.50 (16.1)		57.91 (7.8)		56 (32.4)	-	-
11-15	55 (13.0)	56.31 (14.5)		52.76 (7.6)		-	55 (57.9)	-
16-20	40 (9.5)	55.70 (17.0)		54.55 (8.0)		-	40 (42.1)	-
21-30	81 (19.2)	50.86 (14.3)		55.30 (7.9)		-	-	81 (52.6)
>31	73 (17.3)	47.10 (12.2)		52.12 (8.6)		-	-	73 (47.4)
**Regional Location**, no. (%)								
New England (CT, ME, MA, NH, RI, VT)	22 (5.2)	55.18 (16.9)	.42	51.86 (7.3)	.37	7 (4.0)	3 (3.2)	12 (7.8)
Middle Atlantic (NJ, NY, PA)	98 (23.2)	56.72 (14.4)		55.80 (8.5)		42 (24.3)	23 (24.2)	33 (21.4)
East North Central (IL, IN, MI, OH, WI)	73 (17.3)	53.18 (14.3)		55.75 (8.2)		27 (15.6)	21 (22.1)	25 (16.2)
West North Central (IA, KS, MN, MO, NE, ND, SD)	18 (4.3)	54.83 (12.7)		55.50 (6.3)		7 (4.0)	3 (3.2)	8 (5.2)
South Atlantic (DE, DC, FL, GA, MD, NC, SC, VA, WV)	62 (14.7)	56.11 (14.7)		55.50 (8.6)		24 (13.9)	13 (13.7)	25 (16.2)
East South Central (AL, KY MS, TN)	16 (3.8)	58.81 (12.4)		53.56 (11.2)		7 (4.0)	3 (3.2)	6 (3.9)
West South Central (AR, LA, OK, TX)	28 (6.6)	58.82 (17.4)		56.57 (7.2)		11 (6.4)	6 (6.3)	11 (7.1)
Mountain (AZ, CO, ID, MT, NV, NM, UT, WY)	42 (10.0)	51.60 (15.7)		53.60 (7.4)		22 (12.7)	10 (10.5)	10 (6.5)
Pacific (AK, CA, HI, OR, WA)	61 (14.5)	56.69 (17.1)		53.49 (7.8)		26 (15.0)	13 (13.7)	22 (14.3)
International	2 (.5)	47.00 (14.1)		50.00 (18.4)		-	-	2 (1.3)
**Primary Clinical Setting**, no. (%)								
Acute care hospital	19 (4.5)	63.42 (14.7)	.46	58.16 (6.2)	.69	10 (5.8)	5 (5.3)	4 (2.6)
Hospital-based outpatient facility or clinic	145 (34.4)	54.90 (15.1)		54.40 (7.8)		51 (29.5)	36 (37.9)	58 (37.7)
Private outpatient office or group practice	186 (44.1)	54.89 (15.0)		55.18 (8.5)		85 (49.1)	38 (40.0)	63 (40.9)
Skilled nursing facility (SNF)/long-term care	12 (2.8)	52.00 (14.0)		56.00 (9.1)		4 (2.3)	3 (3.2)	5 (3.2)
Patient’s home/home care	10 (2.4)	55.30 (16.6)		52.40 (8.5)		5 (2.9)	1 (1.1)	4 (2.6)
School system (preschool/primary/secondary)	5 (1.2)	59.00 (12.2)		58.00 (3.1)		3 (1.7)	-	2 (1.3)
Academic institution (post-secondary)	29 (6.9)	56.52 (17.4)		54.38 (9.9)		6 (3.5)	10 (10.5)	13 (8.4)
Health and wellness facility	2 (.5)	57.00 (.0)		51.00 (5.7)		2 (.8)	-	-
Research center	2 (.5)	60.50 (12.0)		52.50 (.7)		1 (.6)	-	1 (.7)
Industry	4 (.9)	52.75 (16.4)		55.25 (9.1)		1 (.6)	-	3 (1.9)
Inpatient rehab facility (IRF)	8 (1.9)	62.00 (16.2)		53.25 (9.4)		5 (2.9)	2 (2.1)	1 (.6)
**CIPS Category**, no. (%)								
Low IP	75 (17.8)					13 (7.5)	17 (17.9)	45 (29.2)
Moderate IP	205 (48.6)					78 (45.1)	46 (48.4)	81 (52.6)
Frequent IP	113 (26.8)					64 (37.0)	23 (24.2)	26 (16.9)
Intense IP	29 (6.9)					18 (10.4)	9 (9.5)	2 (1.3)
**Impostor**, no. (%)*	133 (31.5)					79 (45.7)	30 (31.6)	24 (15.6)

CIPS = Clance Impostor Phenomenon Scale; TAS = Tolerance of Ambiguity Scale, Budner 1962 a Kruskal-Wallis Tests ^ Kruskal-Wallis was attempted, but Mann-Whitney U was completed based on the limited numbers of non-binary and prefer not to answer # Participants were allowed to select all degrees that applied * Impostors were determined by CIPS score of 62 or higher, Holmes et al. 1993

Ethnic data was compressed to White, non-white, or prefer not to say.  A significant difference was noted for the diversity, χ^2^(10, 422) = 301.84, p = .02 of early (14.5%; n = 25 and middle (15.8%; n = 15) compared to late (5.2%; n = 8) career PTs.  There were no significant relationships between career stage and geographical location, clinical setting, or gender. 

Early-career PTs scored significantly higher on the CIPS, H(2, n=422) = 52.17, p<.001 and TAS, H(2, n = 422) = 11.28, p<.01 compared to middle and late-career PTs.  There was a significant difference, χ^2^(6, 422) = 46.19, p<.001 among the IP characteristics of each career stage.  Late-career PTs were more likely to have low IP characteristics (29.2%; n=45), while early-career PTs were statistically more likely to present with frequent to intense IP characteristics (47.4%; n = 82) (Table 1). This distinction was also evident when using the cutoff score for impostor classification, χ^2^(2, 422) = 34.16, p<.001; early-career PTs were statistically more likely to be impostors (45.7%; n=79) than late-career PTs (15.6%; n= 24) (Table 1).

### Correlations and Regression

Age was used as a quantitative proxy variable for respondents’ career stage to assess the strength of relationships between the expressed psychological constructs.  There was a small negative correlation between age and TAS scores, r = -.19, p<.01, 95% CI [-.28, -.09] while there was a medium negative correlation between age and CIPS scores, r = -.36, p<.01, 95% CI [-.44, -.27].  Age accounted for 3.6% of the variance per TAS score and 13% of the variance per IP score.  There was a small positive relationship between CIPS and TAS score, r =.10, p<.05, 95% CI [.004, .190].  A simple bivariate regression between CIPS and TAS was calculated to determine their relationship.  Although the regression equation was significant, less than 1% of the variance is due to the change in score.

### Internal validity of the Scales

The Cronbach’s alpha coefficient was recalculated with all respondent’s data for both outcome measures.  While the internal consistency of CIPS, ⍺ = .93, 95% CI [.92, .94], remained high, the TAS, ⍺ = .48, 95% CI [.40, .55], dropped compared to the pilot data.

## Discussion

The purpose of this study was to assess the prevalence and contextualize the relationship of IP and AT in practicing PTs.  According to the 2019 APTA’s workforce analysis, the median age of licensed PTs was 40, 65% were women, and 84.3% were White.^36^ Although the workforce data was collected before Covid-19 and the data reported here was collected in the spring of 2021 (during the pandemic), respondents’ demographics were similar in regard to age, gender, and ethnicity.  Each geographical location and clinical setting was represented in this study, however, due to the targeted recruitment there were statistically more PTs from the middle Atlantic region and in outpatient settings.  While this may have introduced some bias, the workforce analysis data also demonstrates a higher population of PTs in the middle Atlantic region.

Although some evidence supports significant gender differences in IP rates or AT, most of the studies were limited to a dichotomous gender definition.  In this study most of the respondents identified as women and there were no differences in IP or AT due to gender or ethnicity.  Furthermore, in an attempt to validate IP in managers, it was concluded that professional advancement requires personal characteristics that detract from divergent gender stereotypes, which explains the insignificance of gender differences.^11^ Therefore, exploring managerial and entrepreneurial literature may also be an important next step in this research as many PTs manage clinics within larger institutions or start private practice.  Meanwhile, ethnicity plays an important role in professional advancement and psychological distress, but ethnic differences in IP or AT presentation have been inconsistent in the literature, providing another important area for future research.

Notably, in this study, 31.5% (n=133) of practicing PTs were classified as impostors, similar to reports in healthcare students.^7^^,^^18^^,^^30^   Furthermore, the most prevalent IP characteristics were in the moderate to frequent range.  These data align with a recent study of Indian physiotherapists who demonstrated a similar prevalence of moderate IP characteristics.^37^  However, the physiotherapists were on average 20 years younger than the PTs in this report, most likely due to a Bachelor’s level entrance to clinical practice.^37^  The common IP characteristics of North American healthcare professional students (medical, nursing, dental, pharmacy, chiropractors), nurses, medical residents, physicians, and managers are also similar to the results of this study.^7^^,^^11^^,^^13^^, ^^14^^,^^17^^,^^19^  Researchers suggest that healthcare professionals start to develop feelings of IP when training with other, equally capable students and internalizing any comparisons regarding the value of achievement.^14^^,^^17^   Initial IP feelings are said to remain or even advance as professional training and responsibilities increase.^9^  However, our research demonstrated a reduction in IP feelings with age and experience, similar to a recent study in chiropractic students.^13^  Nearly half (45.7%; n = 79) of the early-career respondents in our study were classified as impostors compared to only 15.6% (n =24) of late-career respondents. These numbers should strike alarm regarding the potential intrinsic barrier of IP when individuals achieve success or take leadership roles in physical therapy practice. These data suggest that the profession would benefit from a longitudinal study aimed to categorize any IP feelings that could lead to attrition of early-career PTs.

Most PTs in our study demonstrated tolerance to ambiguity, which is assumed necessary to manage the complexity of clinical decision-making, autonomous practice, and the “it depends” answers within physical therapy practice. These data add more evidence to the physical therapy graduate questionnaire data (modified from the Association of American Medical Colleges graduate questionnaire) that demonstrate AT comparable to medical students.^28^ Interestingly, the results of this study also provide evidence to the anecdotal assumption that clinicians in inpatient and “other” clinical settings are more tolerant to ambiguity than those in outpatient.

Data from our study also demonstrate that more clinical experience results in lower IP characteristics and more AT.  Early-career PTs, or novice therapists may face feelings of intimidation from the physical therapy workplace.  Difficulty can arise with the dynamic nature of the profession, potentially initiating psychological distress often seen with high levels of IP and intolerance to ambiguity.^5^^,^^24^ Early-career PTs appear more likely to present with IP which may increase their sense of fraudulence, self-doubt, and mistrust in their abilities and accomplishments.  Problem-solving skills developed by experts depend on the individual’s mastery of a particular content domain.^38^ Obtaining the fortitude to push into mastery of skills and success can be a significant hurdle for impostors to overcome.

High levels of impostor fears can lead to decreased job satisfaction, burnout, psychological distress, feelings of self-doubt, and depression.^31^  Success is not an innate feeling for the individual that perceives themselves to be an impostor and may influence decisions for rejecting more professional responsibility, avoiding leadership or managerial roles, or can lead to burnout and attrition.^39^  In our study, there was a significant negative correlation between age and the CIPS and TAS, meaning the older the individual, the less feelings of IP and the more tolerant to ambiguous situations.  Prior research has shown that AT does increase with age ^40^ and that women’s age is inversely correlated with IP.^41^ Considering these data regarding early-career PTs, administrators need to consider authentic opportunities for mentorship and effective strategies to combat the IP characteristics in novice clinicians.^42^

### Limitations

Survey research contributes to well-documented limitations regarding response bias and limited generalizability.  However, the respondents in this study were similar to the demographics of the 2019 APTA workforce analysis and therefore provide a foundation for more targeted PT investigations. During the pilot, the TAS internal consistency was acceptable and better than previously published.^21^  However, when the entire sample was analyzed, the internal consistency dropped, which may provide an opportunity to investigate the single-factor approach to TAS or recognize that several situational factors influenced the outcome scores.^33^  Although using the original TAS may be considered a limitation of this study, it is still the most used^34^ measure for ambiguity tolerance in the literature.^22^^,^^23^^,^^43^  There are no gold standard cutoff scores for the TAS like there are for the CIPS, leaving a vague interpretation of the TAS scores. Furthermore, modifications of Budner’s original TAS are widely used in health professional research, looking at both providers and students.^23^^,^^24^^,^^28^^,^^43^

The targeted recruitment contributed to an imbalanced representation of clinical settings and geographical locations, potentially limiting the generalizability of the results.  Furthermore, specific clinical specialties or certifications were not surveyed.  These events may contribute to unknown bias in our data analysis and warrant more targeted future studies regarding the IP and AT characteristics in practicing PTs and residency or specialty training.

Lastly, the Covid-19 pandemic was a confounding variable that could not be controlled for the duration of this study.  Due to the uncertainty of the pandemic, in addition to consistently evolving work regulations and an increased reliance on telehealth, clinicians’ IP feelings and intolerance to ambiguity could have been exacerbated.  Early-career or novice clinicians may have been impacted the most.  More experienced clinicians may have adapted more readily to a changing Covid-19 work environment.

## Conclusions

The findings of this study conclude that IP characteristics may be frequent or intense as DPT graduates begin clinical practice but more clinical experience may lower IP characteristics and improve AT.  Future research should attempt to discover the underlying reason for these psychological phenomena and explore when levels stabilize.  Meanwhile, there are opportunities to correct and prevent the risk of IP.  Organized mentorship for new graduates by more experienced clinicians, following an educational, progressive plan to build confidence in early-career PTs as they enter the clinical setting, may combat the rise of IP characteristics.^15^^,^^42^

### Acknowledgements

The authors would like to thank Dr. Pauline Rose Clance for granting permission to use the CIPS and Drs. Gabriel Lamm, Matt Pasqualone, Nicolette Weiss, Sarah Maria Berkenstock, Mary O’Malley, and Madison Hess who were the student researchers for this project.

### Conflict of Interest

The authors declare that they have no conflict of interest.
